# A Reply from the New Jersey Secretary

**Published:** 1898-08

**Authors:** 


### A REPLY FROM THE NEW JERSEY SECRETARY.

Upon another page is presented to our readers a rejoinder to our article (May, 1898). The reply speaks for itself and does not require an answer upon our part. If the State of New Jersey and its examining board are satisfied with its representative, the "Secretary, Dental Commission," this journal has no cause for complaint, but it is to be regretted that some one was not found willing to meet the issue made, and who would be qualified to present the subject with the dignity worthy the State and the profession.

The quotations made from various speakers in 1892 may or may not truly represent their opinions at the present time. The gradual encroachments upon the rights of colleges, by State boards and the National Association of Examiners during the past six years, have led many of the teachers in colleges to revise their opinions, and, doubtless, the gentlemen quoted may have something to say upon the subject at the proper time, in fact, one (Dr. Peirce, International Dental Journal, April, 1898) has already given his views, which our correspondent failed to find suitable for his purpose.

The attempt, by implication, to bring Dr. Jack into the circle of defenders of the right to practise irrespective of collegiate training is a violation of the rights belonging to every individual, and will add nothing to the reputation of our correspondent. In justice to Dr. Jack, the following quotations from his paper, read at the meeting alluded to, are given, and they are recommended for the careful reading of the New Jersey State Board.

"The presence of the principle, however, that any person may come forward and submit to an examination has had a demoralizing effect upon many who, without this opportunity, would have had no other course open than either to undergo a college preparation or abandon the attempt. . . . My views upon the next to impossibility of any person of the class indicated making a creditable examination have been long entertained, and have become strengthened by my experience on the examining board of the State. When we come to another class of persons, those who have attended two years of school training, who may be offering themselves ere long in the States which are required to examine any who may present for certificates, the discussion assumes a still more serious aspect.

"The expression of opinions by leading dentists, that the curriculum of the dental schools required enlargement, and the period of study extension, with the concurrent action of the schools and the Association of Dental Faculties to the same effect, resulted in improved courses of study in some of the schools, and the enforcement by the Association of Faculties upon their associates of the three years' term of instruction. . . . The danger of this action being weakened by the obligation of the examining boards in so many States to make examination of any undergraduate who may present for this test makes the question now before us an extremely important one. It is readily perceived that it will tend to weaken the cause of dental education, and is a menace to the three years' course of instruction.

"Examining boards may have an understood policy, which may render it difficult for any one to pass their ordeal, but it appears there can be no safety for the schools of the dental profession until the laws are modified to make it impossible to get a certificate of qualification from a board of examiners alone."

The general tone of the communication from the secretary makes further comment unnecessary. Its tendency will be to still further lower the estimation placed upon the law of New Jersey by a very large number, and will prove, more than any words of ours, that the future well-being of the dental profession does not lie in the direction of boards of examiners governed by laws similar to that recently enacted in that State, nor will the profession be ennobled by the degrading tendencies which it represents.

